# Label-free quantitative proteomics and immunoblotting identifies immunoreactive and other excretory-secretory (E/S) proteins of *Anoplocephala perfoliata*

**DOI:** 10.3389/fimmu.2022.1045468

**Published:** 2022-11-16

**Authors:** Katja Hautala, Jami Pursiainen, Anu Näreaho, Tuula Nyman, Pekka Varmanen, Antti Sukura, Martin K. Nielsen, Kirsi Savijoki

**Affiliations:** ^1^ Veterinary Pathology and Parasitology, Department of Veterinary Biosciences, Faculty of Veterinary Medicine, University of Helsinki, Helsinki, Finland; ^2^ Institute of Clinical Medicine, Department of Immunology, University of Oslo and Rikshospitalet Oslo, Oslo, Norway; ^3^ Department of Food and Nutrition, Faculty of Agriculture and Forestry, University of Helsinki, Helsinki, Finland; ^4^ Department of Veterinary Science, Maxwell H. Gluck Equine Research Center, University of Kentucky, Lexington, KY, United States; ^5^ Division of Pharmaceutical Biosciences, Faculty of Pharmacy, University of Helsinki, Helsinki, Finland

**Keywords:** tapeworm, cestode, horse, E/S proteins, LFQ-proteomics, immunoproteomics, antigen, immunogen

## Abstract

*Anoplocephala perfoliata* is a common tapeworm in horses causing colic and even mortalities. Current diagnostic tests to detect *A. perfoliata* infections have their limitations and an improved method is needed. Immunoreactive excretory/secretory proteins (E/S proteome) of this parasite can provide promising candidates for diagnostic tests. We compared E/S proteins produced by small (length < 20 mm, width < 5 mm) and large (length 20 to 40 mm, width 5 to 10 mm) *A. perfoliata* worms *in vitro* by label-free quantitative proteomics using a database composed of related *Hymenolepis diminuta, Echinococcus multilocularis/granulosus* and *Taenia aseatica* proteins for protein identifications. Altogether, 509 E/S proteins were identified after incubating the worms *in vitro* for three and eight hours. The greatest E/S proteome changes suggested both worm size- and time-dependent changes in cytoskeleton remodeling, apoptosis, and production of antigens/immunogens. The E/S proteins collected at the three-hour time point represented the natural conditions better than those collected at the eight-hour time point, and thereby contained the most relevant diagnostic targets. Immunoblotting using antibodies from horses tested positive/negative for *A. perfoliata* indicated strongest antigenicity/immunogenicity with 13-, 30- and 100-kDa proteins, involving a thioredoxin, heat-shock chaperone 90 (Hsp90), dynein light chain component (DYNLL), tubulin-specific chaperone A (TBCA) and signaling pathway modulators (14-3-3 and Sj-Ts4). This is among the first studies identifying new diagnostic targets and *A. perfoliata* antigens eliciting a IgG-response in horses.

## Introduction

*Anoplocephala perfoliata* is the most prevalent equine tapeworm species and it occurs in grazing horses worldwide (1). It causes pathological changes in its predilection sites, the ileocecal valve and the cecum (2). Equine tapeworm infection has been reported as a cause of various forms of colic (3–5). *Anoplocephala perfoliata* has an indirect lifecycle with oribatid mites acting as intermediate hosts. Horses get infected by ingesting mites while grazing, and larvae develop into adult, egg-producing parasites in the digestive tract without a migration phase (5). The prepatent period takes six to ten weeks (1). The prevalence of *A. perfoliata* may be as high as 60% in some geographic areas (5).

Intestinal parasite infections in horses are routinely diagnosed by use of fecal egg counting techniques. However, this approach can only detect patent infections when the adult worms are producing eggs in the intestinal lumen. Moreover, commonly used fecal egg detection techniques, based on passive flotation, have low sensitivity in diagnosing *A. perfoliata* infections (5, 6). Antibody detection in serum and saliva has been utilized for diagnosing *A. perfoliata* infections (7–9), and antibody-directed enzyme-linked immunosorbent assays (ELISA) for equine sera and saliva are now commercially available for diagnosing *A. perfoliata* infection (5). Horses may, however, remain antibody positives for long periods after the infection (9, 10) and these tests are, thus, unable to differentiate ongoing infection from a recently resolved infection. Polymerase chain reaction (PCR) for amplification of parasitic specific DNA in feces has also been used for *A. perfoliata* diagnostics, but the diagnostic sensitivity of this method is only slightly higher than the egg counting techniques (11). Development of a coproantigen ELISA to detect excretory/secretory (E/S) antigens produced by *A. perfoliata* in equine feces has been reported (12, 13). However, at this point, there are no commercial tests available to diagnose *A. perfoliata* E/S coproantigens. A test based on detecting early E/S antigens could also diagnose prepatent, immature infections, since it is not necessarily associated with egg-production. A reliable test to detect *A. perfoliata* infection would be crucial for reducing unnecessary use of anthelmintics. In addition, detection of this tapeworm as early as possible would allow treatment of infected horses already before the worms start shedding eggs and contaminate pastures.

The E/S proteins excreted/secreted by *A. perfoliata* during infection are the likely key players mediating pathogenesis and host/parasite interaction (14). This protein subgroup forms the first molecular interaction line with the host and, thus, contains promising candidates for diagnostic innovations. Earlier immunoblotting studies have demonstrated that the antibody response to *A. perfoliata* E/S proteins of 12 and 13 kDa in size correlate with parasite infection intensity, but the identity of these proteins have remained unknown (12, 13, 15). A recent in-depth RNA sequencing study on this tapeworm generated the first transcriptome for *A. perfoliata* at its adult stage to support the identification of over 800 E/S proteins produced by this species (16).

The aim of the present study was to quantitatively identify all E/S proteins excreted/secreted by *A. perfoliata* and to screen for immunoreactive E/S proteins for future diagnostic innovations. To meet this goal, we first identified and compared the E/S proteomes generated by *A. perfoliata* worms of different sizes after incubation *in vitro* for different periods of time. Then, we complemented the proteomic results by identifying several potential *A. perfoliata-*associated antigens producing IgG-response in the horse and suggested candidates for accurate diagnostics for *A. perfoliata*. To the best of our knowledge, this is among the first studies providing in-depth knowledge of *A. perfoliata* antigens producing IgG-response in the horse and mediating horse-parasite interaction(s).

## Materials and methods

### Collection and cultivation of *Anoplocephala perfoliata* worms *in vitro*

Live morphologically identified *A. perfoliata* worms and horse sera were collected at an abattoir in Finland (Aug 2019 to Aug 2021). Ethical approval was not needed as no live animals were included in the study and no animals were killed nor harmed due to this study. A written approval from the abattoir was obtained for material collection. Slaughtered horses were inspected for cestode infection. Both cecum and colon of each horse were cut open, intestinal contents removed and *A. perfoliata* worms collected and counted.

**Figure 1:**
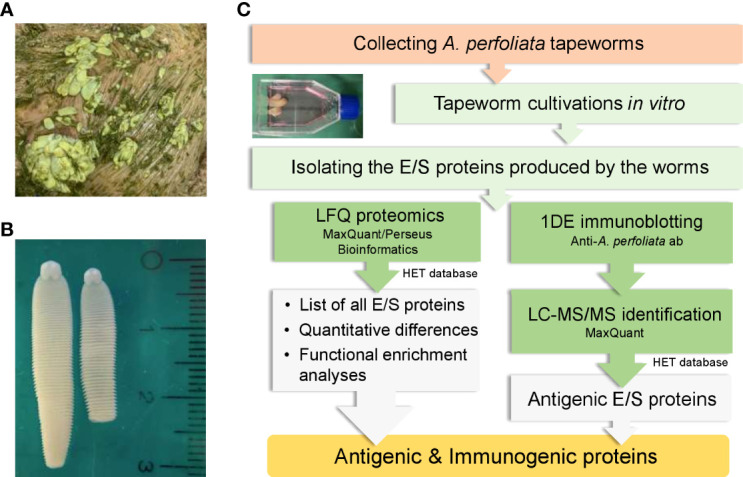
Collecting and culturing *A perfoliata* tapeworms to produce E/S proteins *in vitro* for proteomics and immunoproteomics. **(A)** worms before collection **(B)** macroscopic morphology of *A perfoliata* **(C)** A workflow illustrating different stages used for producing *in vitro* E/S proteins and their quantitative identification and screening for antigenic proteins using horse antisera containing antibodies to *A perfoliata*.

Cultivation of the collected *A. perfoliata* worms and isolation of the E/S proteins produced by the worms was conducted as outlined in **Figure 1**. Briefly, *A. perfoliata* worms originated from two horses: the first had 21 small-size worms (SWs) and from the second, 11 large-size worms (LWs) were collected. The length of SWs was less than 20 mm, and the width was less than 5 mm. The length of LWs varied between 20 to 40 mm and the width between 5 and 10 mm. The small and large worms were handled separately throughout the study. After collection, the worms were washed three times with phosphate buffered saline (PBS) and then once with PBS containing 0.1% *v/v* antibiotic antimycotic solution A5955 (Sigma-Aldrich Oy, Helsinki, Finland). During transportation from the abattoir to the laboratory, SWs and LWs were incubated separately in 100 ml of prewarmed RPMI-1640 medium provided as sterile-filtered liquid with L-glutamine, sodium bicarbonate and D-glucose at 1.8 - 2.2 g/l (Sigma-Aldrich Oy, Helsinki, Finland) at 37°C for three hours. After the three-hour transportation, the culture media were collected for analysis. The worms were transferred to fresh prewarmed RPMI-1640 culture medium for continued cultivation as follows. SWs were divided into four cell culture flasks (volume 40 mL), three flasks containing four worms each and one flask containing nine very small (length < 15 mm, width < 2 mm) worms. LWs were placed into three separate culture flasks with two flasks contained four worms and one flask contained three worms. Each cell culture flask contained 20 mL of RPMI-1640 culture medium. The flasks were incubated at +37°C in 5% CO_2_ for an additional five hours. This additional five-hour incubation only provided three replica samples from SWs and one from LWs, due to loss of viability. Loss of viability was observed in the flasks with the highest number of worms in both experimental groups; one flask with nine SWs and two flasks with four LWs contained only dead worms. Viability of the worms was visually inspected; worms were considered non-viable/dead if they were flaccid with no movement observed. Collected culture media were centrifuged to remove possible debris and bacterial cells (1800*g*, 15 min, +4°C) and then stored at -80°C until used. The E/S proteins produced by the SWs were assigned as E/S_SW_ and proteins related to the LWs as E/S_LW_.

### E/S protein purification and in-solution tryptic digestion

After thawing on ice, the culture supernatants (E/S_SW/LW___3/8h_) were mixed with ice-cold trichloroacetic acid (TCA) in a final concentration of 4% *w/v* and the suspensions were incubated overnight on ice. Proteins were precipitated by centrifugation (8000*g*, 30 min, +4°C), washed twice with cold acetone (-20°C) and air-dried for few minutes. Precipitated proteins were solubilized in 0.1% *w/v* RapiGest™ SF (Waters Corporation, Milford, MA, USA) in 100 mM triethylammonium bicarbonate (TEAB, pH 8.5). The protein concentration was measured using the NanoDrop™ 2000/2000c Spectrophotometer (Thermo Fisher Scientific, Vantaa, Finland). For tryptic digestions, the proteins (10 µg) were first reduced with 10 mM dithiotreitol (DTT) in 100 mM TEAB for 45 min at +60°C, then alkylated with 15 mM iodoacetamide (IAA) in 100 mM TEAB for 60 min at room temperature (RT) in the dark and finally quenched with 20 mM DTT in 100mM TEAB. Trypsin (mass-spec grade, Promega) was added to each sample at a ratio of 1:50 (trypsin: protein ratio, *w/w*) and the reactions were incubated overnight at +37°C. Digestions were stopped by adding trifluoroacetate (TFA) (Sigma-Aldrich Oy, Helsinki, Finland) to a final concentration of 0.6% *v/v* and the peptides were purified using ZipTip C18 (Millipore) and air-dried. Since RPMI-1640 used for culturing the worms contains no proteins nor peptides, there was no need to perform LFQ proteomics on the culture medium to subtract possibly interfering proteins or peptides from the proteomic analyses.

### Label-free quantitative proteomics

The peptides (5 µg) reconstituted in 0.1% *v/v* formic acid (FA) were analyzed with an Easy-nLC 1000 nano-LC system (Thermo Scientific, Waltham, MA, USA), coupled with an Orbitrap Q Exactive Plus quadrupole mass spectrometer (Thermo Scientific, Waltham, MA, USA) equipped with a Nanoelectrospray Ion Source (Easy-Spray, Thermo Scientific, Waltham, MA, USA). The LC separation was performed using a C18 column (25-cm bed length 2-μm beads, 100 Å, 75-μm inner diameter) with a flow rate of 300 nl min^-1^. The peptides were eluted with a 2%–30% solvent gradient (100% acetonitrile/0.1% FA) over 60 min. The MS was operated in the data-dependent acquisition mode, with the 10 most abundant multiple-charged ions selected for fragmentation. The obtained MS raw files were submitted to the MaxQuant software v.1.6.7.0 (17) for protein identification and label-free quantification (LFQ) using a protein database (HET) combining all protein sequences from the following other cestodes; *Hymenolepis diminuta* (Proteome ID, UP000321570, 18691 protein sequences), *Taenia aseatica* (Proteome ID, UP000046400, 10327 protein sequences) and *Echinococcus granulosus/multilocularis* (Proteome IDs, UP000019149/UP000019149, 34099 protein sequences) (18–20). These cestodes were selected as neither whole genome sequence nor the protein sequences based on the published *A. perfoliata* transcriptome (16) to support proteome-wide studies have been released. Only the mitochondrial genome to support phylogenetic studies on this tapeworm is available (21, 22). *Echinococcus multilocularis/granulosus* and *H. diminuta* are shown to share the closest gene homologs to their counterparts in *A. perfoliata* (16). Mass tolerances of 20 ppm and 4.5 ppm were applied for the first and main search, respectively. Trypsin digestion without proline restriction option was applied with two missed cleavages allowed. The minimal unique + razor peptide number was set to one, False Discovery Rate (FDR) to 0.01 for peptide and protein identification, and a label-free quantification (LFQ) with default settings was performed (23).

### Proteome bioinformatic analyses

Perseus v.1.6.1.3 and v. 1.6.2.3 were used for pairwise co-comparisons and multivariate analyses (24). Principal component analysis (PCA) was carried out with un-normalized raw intensity and normalized LFQ intensity values. For hierarchical clustering analysis, the normalized LFQ values were log2 transformed, imputed by random draws from the normal distribution (width = 0.3, down shift = 1.8) and z-score normalized with proteins identified in at least 3/4 replicates and in at least one sample group. Two-sample *t*-test with a permutation-based FDR adjustment was used on log10-transformed LFQ intensity values to indicate statistically significant protein abundance changes. The STRING database v. 11 (25) was used to indicate protein-protein interactions, enriched functional pathways and biological processes (InterPro, KEGG, Gene Ontology) within the selected E/S protein sets, with following parameters: full network, meaning of network edges set to evidence and interaction score set to high confidence (0.700). The interacting proteins were clustered using the Markov clustering – MCL (26) with the inflation parameter of 4.

### One-dimensional gel electrophoresis and 1DE-immunoblotting

E/S proteins (20 µg) produced by SWs after three hours of incubation were denatured in 1xLaemmli buffer (27) 95°C for 10 min. Proteins were separated in an 8-16% Mini-PROTEAN TGX Stain-Free Precast Gel (Bio-Rad Laboratories, U.S.A.) using 1 x TGS (25 mM Tris, 192 mM Glycine and 0.1% *v/v* SDS) as a running buffer and broad range blue-stained protein markers (10-250 kDa, Precision Plus Protein Standards, Bio-Rad Finland Oy, Helsinki, Finland) as a molecular weight standard. 1DE electrophoresis was performed at 120V constant voltage for 55 min and proteins were visualized by SimplyBlue™ SafeStain according to the instructions provided (ThermoFisher Scientific, Waltham, MA, USA). The proteins were transferred after 1DE onto a polyvinyl difluoride (PVDF) membrane using the Trans-Blot Turbo Transfer System (Bio-Rad Finland Oy, Helsinki, Finland), according to the manufacturer’s instructions (30 min, 1.3 A, 25 V). After electroblotting, the PVDF membrane was treated with SuperBlock^®^ Dry Blend Blocking Buffer (Pierce Biotechnology, Rockford, USA) for 60 min at RT (shaking) to prevent nonspecific antibody binding. Then, the membrane was probed using mixed sera combined from two horses with *A. perfoliata* infection (diluted 1:100 to SuperBlock^®^ blocking buffer). Anti-*A.perfoliata* horse sera was collected from a 10-year-old mare harboring over 500 tapeworms and from a 13-year-old stallion harboring four tapeworms. In addition, three *Parascaris* spp. worms were found inside the stallion’s small intestines. Sera collected from both of the tapeworm infected horses were confirmed positive for *Anoplocephala* IgG antibodies using ELISA test at a commercial laboratory (Austin Davis Biologics laboratory, UK). The control immunoblot was prepared by treating a replicate membrane with the same proteins with three individual antisera (diluted 1:100 to SuperBlock^®^ blocking buffer) collected from three separate horses not infected with *A. perfoliata*. Both cecum and colon of these horses were cut open, intestinal contents removed, and no parasites were observed by macroscopic inspection. Infection-naïve sera were confirmed to have very low titers (close to zero, considered negative) for *Anoplocephala* IgG-antibodies at a commercial laboratory (Austin Davis Biologics laboratory, UK). Two of the infection negative horses were geldings and one was a stallion, their ages varied between five to thirteen years. Each membrane with the indicated horse serum was incubated overnight at +4°C (gentle shaking). Sera were collected during the normal slaughtering process from worm positive/negative horses.

After exposure to the indicated horse serum, the PVDF membrane was washed five times with PBS containing 0.1% (*v/v*) Tween 20 at RT (vigorous shaking) and then subjected to 1:1000 diluted rabbit anti-horse IgG, conjugated with Horseradish peroxidase (Nordic-MUbio, BC Susteren, Netherlands), in SuperBlock^®^ blocking buffer. After 60 min incubation at RT (gentle shaking), the blots were washed five times with PBS containing 0.1% (*v/v*) Tween 20 at RT and finally the Tween 20 was removed by rinsing the membrane in PBS. The blots were visualized with Pierce DAB Substrate Kit (ThermoFisher Scientific, Waltham, MA, USA) and the antigenic protein bands were subjected to relative quantification using the AlphaView software (AlphaImager HP system, version 3300, Alpha Innotech. Corp.). For identification, the corresponding protein bands were cut out from the gel stained with SimplyBlue™ SafeStain followed by in-gel tryptic digestion and LC-MS/MS analysis of the resulting peptides (27) sing timsTOF fleX (Bruker) coupled to nanoElute (Bruker). The obtained LC-MS/MS files were searched with MaxQuant as described above. The most promising antigens in each protein band were then listed according to the following criteria: the high identification intensity value of the detected proteins, the predicted molecular weight of the identified protein correlating to the observed molecular weight for the corresponding protein band and the previously reported antigenicity/immunogenicity for the identified protein. Immunoblotting analyses with combined antisera tested positive for *A. perfoliata* IgG-antibodies were repeated at least three times. Immunoblotting analyses with horse sera tested negative for *A. perfoliata* were conducted separately with serum collected from three individual horses.

## Results

### A total of 506 individual E/S proteins were excreted/secreted by *A. perfoliata in vitro*

**Table S1** lists a total of 673 proteins excreted/secreted by SWs and LWs. The quality of each data set without prior filtering was good; a broad overlap in protein identifications was detected within all four biological replica samples; ~50-77% of all identified proteins were shared by each replicate and ~70-90% by three replicates involving the E/S identification datasets at three-hour time point. At the eight-hour time point three SWs- and one LWs-associated protein identification data sets were combined (hereafter SLWs) due to the apparent viability loss observed with the worms at this incubation stage.

The HET protein database, composed of *H. diminuta*, *E. multilocularis/granulosus* and *T. aseatica*, used for E/S protein identifications allowed detection of protein homologs present in one or more of the cestode species. Since the worms were cultured without an antibiotic, preventing possible bacterial growth, only those proteins with high sequence coverage and with several matching peptides were considered as genuine E/S proteins excreted/secreted by *A. perfoliata*. **Table S2** lists proteins that were detected with the highest raw intensity values in at least one of the parasitic species and with at least three out of four replicate samples in any of the identification data sets. The number of remaining individual E/S proteins produced after three and eight hours of incubation *in vitro* and fulfilling these criteria was 506.

### E/S protein excretion/secretion is most efficient by large-size worms

**Figure 2:**
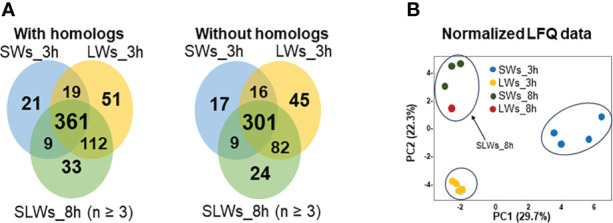
**(A)** Venn diagrams comparing the number of shared and unique E/S proteins (with/without the protein homologs) produced by the SWs, LWs and SLWs after three or eight hours of incubation at 37°C. SWs = small-size tapeworms, LWs = large-size tapeworms, SLWs = combined identification list containing three SWs-associated E/S protein datasets and one LWs-associated E/S protein dataset. **(B)** PCA investigating the relationships between the protein abundance patterns between the indicated E/S proteomes at three- and eight-hour time points.

Venn diagrams in **Figure 2A** compare the number of proteins with/without the protein homologs at the three-hour and eight-hour time points using the HET protein database. In both cases, the greatest number of uniquely identified proteins were produced by LWs (51 with orthologs and 45 without orthologs) after three hours and SLWs (33 with orthologs and 24 without orthologs) after eight hours of incubation *in vitro*. E/S products produced by SWs at the three-hour time point included a total of 343 individually identified proteins. Among these, the top 25 detected with the highest raw intensity values were identified as cytoskeleton-associated components (actin, tubulin α and β chains, dynein light chain and annexin), three glycolytic moonlighters (glyceraldehyde-3-phosphate dehydrogenase - GaPDH, phosphoenol pyruvate carboxykinase - PYK and enolase - ENO), stress-related proteins (glutathione S-transferase, heat-shock chaperones and a proteasome α subunit), a signaling pathway modulators 14-3-3 and nine proteins with yet uncharacterized function (**Table S1**).

### The greatest abundance changes associate with small molecular weight proteins

**Figure 3:**
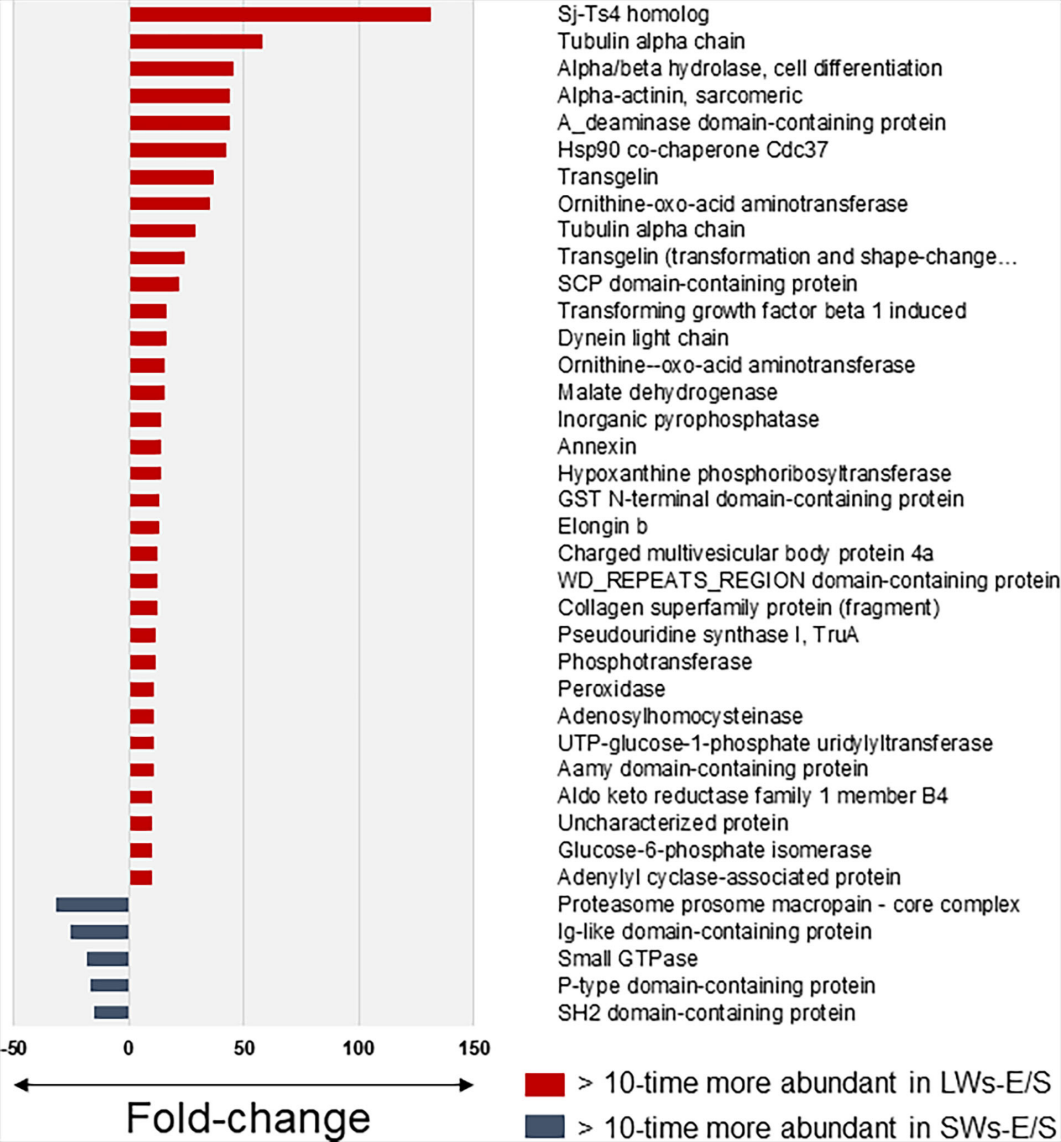
The most significant protein abundance fold-changes between the small (SWs) and large tapeworms (LWs) associated E/S proteins at three-hour time point *in vitro*. The log10 transformed LFQ data was analyzed using Student’s t-test with permutation-based FDR adjustment.

**Table S3** lists a total of 75 proteins with more than two times (*t*-test with permutation-based FDR) higher abundancy in the LW- than the SW-associated E/S proteome at the three-hour time point, whereas only 25 proteins fulfilling this criterion were more abundant among the E/S proteins produced by SWs. **Figure 3** compares the E/S proteins showing >10-times higher abundance difference between the SW- and LW-associated E/S proteomes. Thirty-five proteins were detected as more abundant within the LW-associated proteomes, while only five proteins were more produced by SWs. Among these, a homolog of *Schistosoma japonicum* Sj-Ts4 antigen (28) was detected with the greatest fold-change; this antigenic/immunogenic protein was ca. 130-times more efficiently excreted/secreted E/S protein by the LWs compared to SWs after three hours of incubation. Other remarkably more abundant proteins included tubulin α chain, α/β hydrolase involved in cell differentiation, α-actinin, a deaminase and a heat-shock protein 90 (Hsp90), each with >40-times higher abundance change in LW-associated E/S proteome at three-hour time point compared to these proteins produced by SWs at the same time point. The greatest fold-change among the SW-associated E/S proteins was detected for a proteasome prosome macropain subunit, which was detected with nearly 30-times more efficiently produced by this tapeworm-type compared to LWs after three hours of incubation *in vitro*.

**Figure 4:**
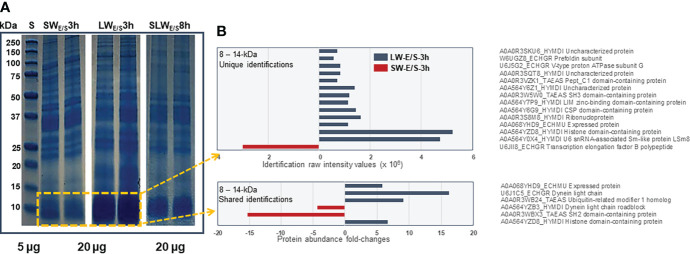
**(A)** Representative 1DE protein profiles of all E/S proteins produced by SWs and LWs at three-hour time point and SLWs after eight hours of incubation *in vitro.* Marker, protein standard (5 µg) with molecular weights between 250 – 10 kDa. **(B)** The most significant protein abundance fold-changes between the SWs- and LWs-associated E/S proteins migrating with molecular weights between 8 and 14 kDa.

**Figure 4A** shows representative 1DE-protein profiles of the E/S proteins excreted/secreted by SWs and LWs at the three-hour time point and SLWs after eight hours of incubation. The most distinct abundance change was linked with ca. 10-kDa protein band (ca. 1.4-times) within the LW- (relative intensity: 52.5 ± 2.4) than the SW-associated (relative intensity: 35.0 ± 5.6) E/S protein profile. **Figure 4B** indicates the raw intensity values for uniquely identified proteins with predicted molecular weights around 10 kDa and LFQ comparison of proteins that were commonly detected within the LW- and SW-associated E/S proteins. The highest raw intensity values associated with histone domain containing protein and U6 snRNA-associated Sm-like protein (LSm8) within the LW-type E/S proteins at 3-hour time point and transcription elongation factor B in SW-type E/S proteins at the same time point. The LFQ comparison indicated a non-catalytic accessory component of the cytoplasmic dynein 1 complex (dynein light chain – DYNLL) with the greatest abundance change (>15-times more produced by LWs than SWs) within this protein band. Taken together, these findings indicated that the E/S proteins migrating around and slightly above 10 kDa were more efficiently produced/excreted/secreted by LWs than SWs after three hours of incubation.

### Majority of the E/S proteome differences are linked with homeostasis and cytoskeleton remodeling

To uncover possible correlations within the identified E/S protein datasets, the normalized LFQ identification data were next compared by principal component analysis (PCA). A principal component analysis in **Figure 2B** explained ~50% of the total variance with clear clustering of each replicate samples at the three-hour time point of incubation. At the eight-hour time point, the SWs- (three replicates) and LWs- (one replicate) associated E/S proteins clustered closer together. The PC1 (variance 29.7%) separated the E/S proteomes according to worm size and PC2 (variance 22.3%) according to the protein abundance changes. Thus, PCA confirmed that the detected E/S proteomes differed between the two worm types at the three-hour time point, and that closely resembling E/S proteomes were generated by SLWs (three E/S protein replicates associated with SWs and one replica with LWs) after eight hours.

**Figure 5:**
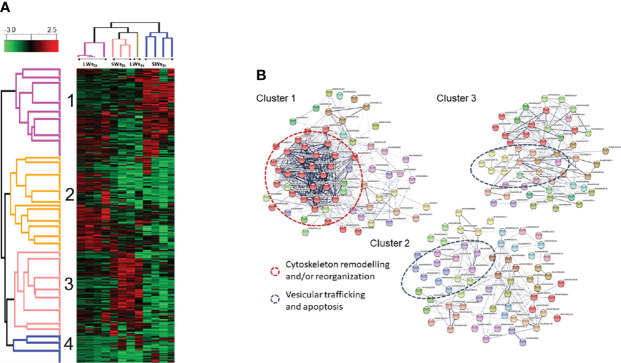
**(A)** Hierarchical k-means clustering of E/S proteins based on their normalized LFQ abundances. Parameters used: Distance metric, L1; Linkage rule, average; Constraint, none. LWs_3h_ = large-size tapeworms at three-hour time point, LWs_8h =_ large-size tapeworms at eight-hour time point, SWs_3h_ = small-size tapeworms at three-hour time point, SWs_8h_ = small-size tapeworms at eight-hour time point (**Table S4**). **(B)** ​An additional STRING PPI analysis with MCL clustering indicates enriched protein-protein interactions and biological functions (**Table S5**).

To obtain a quantitative view of the worm size- and incubation time-dependent effects on the E/S proteomes, the normalized LFQ identification data were also subjected to hierarchical clustering. The dendrogram-heat map in **Figure 5A** indicated four clearly distinct co-abundance protein profiles; **(i)** cluster 1 with proteins more produced by LWs and/or SWs at three-hour time point, **(ii)** cluster 2 containing more abundant proteins only in LW-associated E/S proteomes at the three-hour time point, **(iii)** cluster 3 showing proteins more abundant only in SLWs-associated E/S proteomes after additional five hours of incubation (eight-hour time point), and **(iv)** cluster 4 containing proteins with similar co-abundance pattern in each E/S data set. These results and the clustered proteins are further listed in **Table S4**. An additional STRING protein-protein interaction (PPI) analysis of the clustered proteins **(** **Figure 5B** **)** indicated enrichment of following proteins/protein activities: **(i)** clusters 1 and 2 with cytoskeleton remodeling/reorganization (actin filaments and microtubules, actin folding-related chaperone and proteasome activities), **(ii)** cluster 3 with vesicular trafficking and apoptosis-related activities, and **(iii)** cluster 4 having protein folding/reassembly and degradation-associated activities (**Table S5**). Thus, majority of the apoptosis/stress-related activities within the E/S proteins related to the LW-type tapeworms after three and eight hours of incubation *in vitro*.

### Immunoproteomics identified several potential antigens of *A. perfoliata*

**Figure 6:**
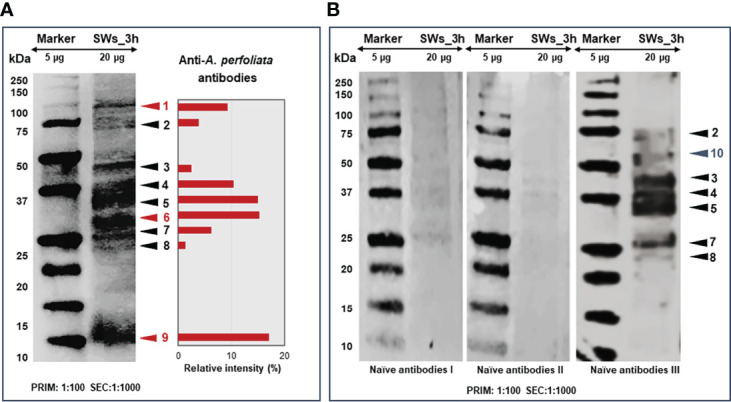
Representative 1DE immunoblots showing the protein bands reacting with the anti-*A. perfoliata* antibodies **(A)** and with antibodies from infection-naïve horse **(B)**. The E/S proteins excreted/secreted by SWs at three-hour time point were electroblotted to PVDF membrane first probed with 1:100 diluted anti-*A. perfoliata* or infection-naïve antibodies and then with 1:1000 diluted rabbit anti-horse IgG (horseradish peroxidase conjugated) followed by DAB staining to visualize the reacting proteins. Arrow heads and numbers in red and black refer to the antigenic/immunogenic proteins specific to *A perfoliata* and other potential parasites and/or micro-organisms, respectively. The cross-reacting protein antigens/immunogens were quantitated using the AlphaView software to obtain relative intensity values (%) for the indicated protein bands.

Since our findings suggested more efficient E/S protein excretion/secretion with SWs at the three-hour time point and increased apoptosis-related activities with LWs and SLWs, the SW-associated E/S proteins at the three-hour time point were screened for antigenic proteins. **Figure 6** shows the E/S antigen profiles after immunoblotting with *A. perfoliata* -infected (A) and infection-naïve (B) horse sera. Altogether, nine protein bands reacted with the anti*-A. perfoliata* IgG-antibodies and, unexpectedly, six with one of the infection-naïve horse serum. Immunoblotting with two naïve horse sera indicated only faint protein bands migrating with molecular weights of ca. 25, 37 and 40 kDa. Three antigenic protein bands (no. 1, 6 and 9), migrating with molecular weights of ca. 13, 30 and 100 kDa were specifically detected only with the anti-*A. perfoliata* antibodies, among which the strongest intensity was obtained with the 13-kDa antigenic protein band **(** **Figure 6A** **)**.

Next, all antigenic protein bands, reacting with both the *A. perfoliata* and infection-naïve antibodies were subjected to in-gel tryptic digestion and LC-MS/MS analysis (**Table S6**). **Table 1** lists the most promising antigens in each protein band. As a result, we determined that a heat-shock protein 90 (Hsp90 in band no. 1), two signaling pathway modulators (14-3-3 and/or Sj-Ts4-like), a dynein light chain component (DYNLL), tubulin-specific chaperone A and/or thioredoxin (protein band no. 9) were only detected with anti-*A. perfoliata* horse sera. The antigenic protein bands detected with both the anti-*A. perfoliata* and the infection-naïve antisera were identified to contain two glycolytic and adhesive moonlighters, such as fructose-bisphosphate aldolase (FBA) and glyceraldehyde-3-phosphate dehydrogenase (GaPDH) with the highest raw intensity values (protein band no. 5), which implies that the horses may have been exposed for example to other parasites than *A. perfoliata*. Only one protein band (no. 10) with molecular weight between 50 and 75 was specifically detected with infection naïve antibodies. This band included many proteins, from which the Heat shock cognate protein 70 (Hsp70; W6U0E5_ECHGR) was identified with the highest raw intensity values (28920) and the highest number of the matched peptides (10).

**Table 1:** List of the E/S protein bands cross-reacting with the anti-*A. perfoliata* and infection-naïve antibodies.

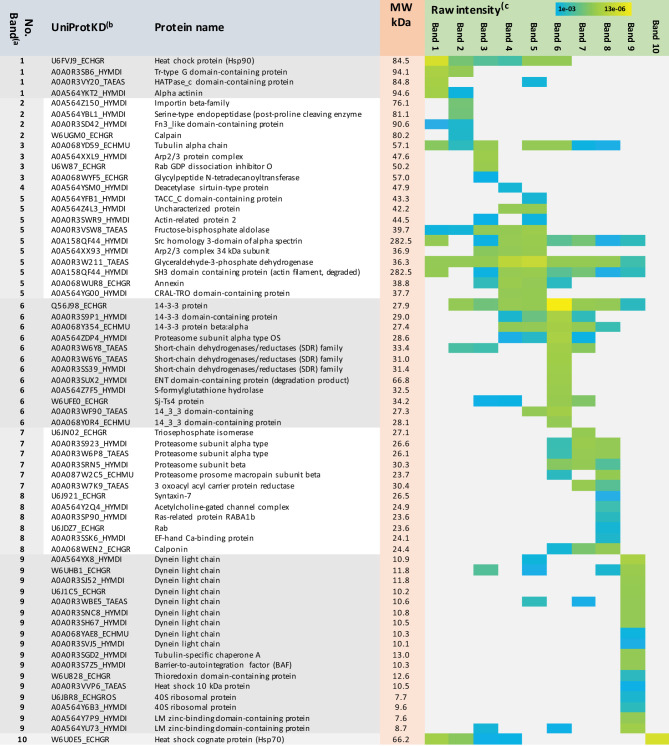

a) Bands were cut out from the replica 1DE gel, in-gel digested with trypsin and b) identified by LC-MS/MS using the HET protein database. c) Raw intensity values, with color gradient corresponding to raw intensity values of the protein, and the number of matched peptides is shown. Cells in grey, protein not detected. No. band, protein band in **Figure 6**.

Shaded band numbers refer to proteins identified from the antigenic protein bands that were specifically detected with anti-A. perfoliata antibodies.

## Discussion

The present study reports in-depth analysis of the *A. perfoliata-*associated E/S proteome and the associated immunoproteome with promising target diagnostic proteins for further studies. Our research demonstrates an association between E/S proteome composition and the *A. perfoliata* worm size as well as the incubation time; the large-size worms (LWs) produced higher number of proteins than the worms of smaller size (SWs) and higher number of proteins were excreted/secreted at the eight-hour time point compared to the earlier (three-hour) time point. Although the length of the worms varied with their movement, the width of the worms was easy to evaluate. The worms were collected from two horses; all SWs from one individual horse and LWs from the other one. The fact that one horse was harboring smaller size worms than the other may indicate shorter infection duration, however, also host specific factors such as its immune response may have influence on worm size.

Our findings indicated that the RPMI-1640 medium used for culturing the worms, the number of worms cultured under the same conditions and/or using a several-hour incubation period might have necessitated further optimization. This was based on the apparent loss of viability associated with conditions involving high number and size of the worms. Here, the proteomic findings indicated that metabolic pathways associated with cell death/apoptosis were activated in LWs at the three-hour-sampling point and in both, SWs and LWs, at eight hours of incubation. Although these findings are in line with those reported by Wititkornkul et al. (16); both the transcriptomic and proteomic results indicated upregulation/increased abundancy of calpain that is a modulator protease closely linked with apoptosis (28, 29), we cannot exclude the possibility that also the *in vitro* conditions used have triggered the death of the worms. Thus, it remains to be shown if refreshing the culture medium more frequently, supplementing RPMI-1640 with some growth stimulating factors (*e.g*., fetal serum albumin) and/or increasing the concentration of D-glucose could have conferred increased viability to the *A. perfoliata* worms.

The previous E/S proteome study on the adult stage of *A. perfoliata* indicated that this organism excreted/secreted 842 individual E/S proteins, and that nearly 60% of these proteins were associated with extracellular vesicles (EVs) (16). EVs are phospholipid bilayer enclosed particles delivering lipids, proteins, nucleic acids, carbohydrates, and metabolites in a concentrated and protected manner into the host for intracellular communication/immunomodulation (30–34). Many identified proteins here were identified as EV-associated proteins by Wititkornkul et al. (16). Thus, it is more than likely that we also detected EV-associated proteins, since these structures are prone to be disrupted during TCA precipitation. On the other hand, our study identified approximately 300 fewer E/S proteins. Possible reasons explaining the variation in the total number of E/S protein identifications in these two studies include the different incubation media and the lack of antibiotics in our media, growth temperature and environment (37°C versus 39°C, 5% CO_2_ versus ~20% atmospheric O_2_) and/or the physiological state and the number of worms used per one culture experiment. We also demonstrated that apoptosis and stress-related pathways were activated in LWs at the three-hour time point and in both the SWs and LWs at eight hours, implying that the *in vitro* conditions triggered these activities and that LWs responded earlier than the SWs. Activation of these pathways has been shown to be linked to increased protein release by vesiculation, which may also explain why a higher number of E/S proteins were excreted/secreted here by LWs and SLWs compared to SWs (16). Indeed, we detected two proteins, a protein implicated in endocytosis, vesicle transport and signal transduction and a ferlin involved in vesicle fusion events as more abundant in LWs- and SLWs-associated E/S proteomes compared to those excreted/secreted by SWs (**Table S3**). A positive correlation between a rise in vesicle production and cell death levels in response to an antibiotic treatment has also been reported with cell lines, and that even low doses of antibiotics may increase vesicle production and traffic (35–37). Thus, to avoid possible antibiotic-induced effect on the protein excretion/secretion, we decided to incubate *A. perfoliata* in RPMI-1640 without antibiotics, which may partially explain why a lower number of E/S proteins were detected.

To screen for the most suitable diagnostic markers we selected the E/S proteins collected from SWs at the three-hour time point. Our findings suggested that apoptosis/stress-related pathways were activated in LWs already at this time point and therefore we assumed that the E/S proteins collected from SWs at the earliest time point would represent the natural conditions best. The most abundant proteins associated with this E/S protein group were identified as actin, tubulin α/β, dynein light chain as well as glycolytic and stress-related moonlighters (GaPDH, PYK, ENO and Hsp70), from which the first three have functions in cytoskeleton remodeling/reorganization. The dynein light chain was also reported among the most efficiently expressed genes by Wititkornkul and co-workers (16). Other highly abundant proteins identified by that study included GaPDH, PYK, ENO and Hsp90, which were also identified here with reasonably high abundancies. These proteins are classified as known moonlighting proteins, as besides their primary roles in glycolysis or stress, they also have a secondary extracytosolic function, for example by showing adherence to the host proteins (38). We also detected a potential glutathione S-transferase, proteasome α subunit and 14-3-3 signal pathway regulator as potentially new moonlighting proteins at high abundancies, which was not observed by Witikornkul and co-workers (16). We suggest that, in addition to the incubation medium and conditions used for *in vitro* incubations, the discrepancies between our and the recently reported E/S proteomic data can partially be explained by the proteomic method used for protein identifications. Wititkornkul and co-workers applied 1D gel electrophoresis followed by Coomassie Blue staining, in-gel tryptic digestion and LC-MS/MS identification (16), while our study was based on in-solution tryptic-digestion of E/S proteins followed by LFQ identification. Both proteomic approaches have pros and cons; for example, the gel-based method, while enhancing the identification of complex/hydrophobic proteins, involves several washing steps prior to in-gel tryptic digestion, which may increase the likelihood of washing out of proteins. The non-gel-based proteomics provides the whole proteome for LC-MS/MS analysis by in-solution tryptic digestion of all proteins in only a single step, which may favor the identification of highly abundant proteins. Thus, besides confirming and complementing the recently reported gel-based proteomic study, we also provide new findings for maximizing the efforts to control *A. perfoliata* infections in horses.

A coproantigen ELISA to detect *A. perfoliata* infection has been based on using a rabbit anti-tapeworm E/S antigen 12/13 kDa IgG polyclonal antibodies (12, 13). In this study, we identified several potentially antigenic/immunogenic E/S proteins, including also the previously detected small-size E/S proteins migrating at 12-13 kDa (7, 12, 13). It has been shown that serum antibodies from individual horses infected with *A. perfoliata* do not always bind to the same E/S antigens produced by this tapeworm, as evidenced by varying immunoblotting results (8). Here, the immunoblotting analyses with antiserum tested positive for *A. perfoliata* IgG-antibodies indicated several possibly antigenic/immunogenic proteins to *A. perfoliata*. However, it should be noted that this preliminary data is not confirmed tapeworm specific and cross reactivity, especially with other common horse parasites, needs to be tested before diagnostic applications. One of the control immunoblots with horse serum that was tested negative for *A. perfoliata* antibodies resulted also several cross-reacting antigens. This finding may indicate earlier exposure to this tapeworm or to other parasitic and/or microbial organism(s). Therefore, the antigens detected with both the anti-*A. perfoliata* and one of the control antisera were excluded from further analyses. Here, we noticed dynein light chain protein homologs (DYNLL) in all three comparison parasite species (*H. diminuta*, *E. multilocularis/granulosus* and *T. aseatica*) with predicted molecular weights of 10 to 12 kDa; DYNLL has been shown to be efficiently expressed both at the gene and protein levels in *A. perfoliata* (16). This component of the cytosolic dynein complex has been reported to regulate the mitochondrial pathway of apoptosis by determining the complex formation and posttranslational stability of the proapoptotic Bcl-2 family proteins Bmf and Bim (39, 40). Other likely *A. perfoliata* and small-molecular weight antigens were identified as a 12-kDa thioredoxin and a tubulin-folding protein TBCA involved in the early step of the tubulin folding pathway. Higher molecular-weight antigens/immunogens specifically detected with anti-*A. perfoliata* antibodies included the signaling pathway modulators 14-3-3 and Sj-Ts4-like proteins. One study has suggested that *A. perfoliata* could export the 14-3-3 protein *via* EVs (16). This protein is also expressed in both the adult and cysticercoid stages of *H. diminuta* (41). In *E. granulosus*, the 14-3-3 signaling protein has been shown to contribute to various cellular functions, including energy production, carbohydrate metabolism, and intracellular trafficking (42). From these two signaling proteins, the Sj-Ts4 was remarkably more produced; ca. 130 times by the LWs compared to the SWs after three hours of incubation. A Sj-Ts4-like homolog has also been identified among the E/S proteins produced by *H. diminuta* adult worms (41) and pig tapeworm, *Taenia solium* (43). Initially, the Sj-Ts4 protein was recovered as a novel 23-kDa antigenic protein of blood fluke, *S. japonicum* (44). This antigen has been localized to the tegument and reported to show high sensitivity and specificity for the diagnosis of *S. japonicum* to aid serological diagnosis of this parasite in endemic areas (45, 46). Here, the identified Sj-Ts4 protein was detected from the protein band no. 5 migrating with molecular weight of around 30 kDa. Sj-Ts4-like protein is a highly promising diagnostic marker for *A. perfoliata* infections in horses, as this protein has only been identified in few flatworm species, among which *A. perfoliata* is the only one infecting horses (16, 41, 44, 47, 48). Being probably a tegument protein also in *A. perfoliata*, one might suspect it to be shed constantly from the worm to the intestinal contents.

Wititkornkul et al. (16) presented three novel *A. perfoliata* proteins from an adult worm; sigma class glutathione transferases - GSTs, Hsp90 and ENO, which were also detected in our study after the LFQ identification. Our immunoproteomic analysis proposes that from these three proteins only Hsp90 could act as an *A. perfoliata*-specific antigen/immunogen. Hsp90 is a widely conserved molecular chaperone within eukaryotes and prokaryotes, which facilitates the maturation of substrates (kinases, transcription factors, steroid hormone receptors and E3 ubiquitin ligases) involved in many different cellular pathways and infectious diseases (49). ENO is classified as an adhesive moonlighting protein with primary role in glycolysis (38) and with reported antigenicity for *H. diminuta* (50). Our immunoblotting analysis suggests that other glycolytic moonlighters FBA and GaPDH, cross-reacting with both the anti-*A. perfoliata* and infection-naïve horse sera, could represent antigens/immunogens to other organisms. This is supported by earlier studies reporting that both moonlighters have been identified as antigenic in *H. diminuta* (50, 51).

The immunoproteomic approach used in the present study was based on the separation of the E/S proteins by 1DE, which does not allow pinpointing the individual protein(s) with antigenic/immunogenic potential, as several proteins can migrate with similar molecular weight(s). An optional method, two-dimensional gel electrophoresis (2DE), suffers from certain limitations, such as the inability to maintain solubility of certain group of proteins (hydrophobic/alkaline/complex proteins) during isoelectric focusing (IEF) – the first step of 2DE (52–58). Therefore, we applied 1DE-based protein separation coupled with immunoblotting to identify as complete immunoproteome as possible, including also the highly alkaline/hydrophobic and complex proteins.

Wititkornkul et al. (16) published a quantitative RNA sequencing analysis performed on the mRNAs isolated from *A. perfoliata* to create a protein database based on the gene expression data for the follow up proteomics. However, neither the RNA sequences nor the protein database(s) are yet available. Defining the genetic code for all *A. perfoliata* genes, not only for those expressed *in vitro*, would also allow more accurate sequence comparisons to pinpoint antigens/immunogens that are specific to cestodes and *A. perfoliata*. Nevertheless, in the future the antigenic proteins proposed in this study should be further tested and analysed for their sensitivity and specificity.

## Conclusions

This study reports in-depth E/S proteome analysis of *A. perfoliata*, which confirms and complements the recent transcriptomic and gel-based proteomic analyses on the proteins released by this tapeworm (16). In addition, our proteomic data provides new information of how the size of the worms affect E/S protein production as well as how this parasite responds to *in vitro* conditions. The most promising targets include a novel heat-shock protein Hsp90, signaling pathway modulators Sj-Ts4 and 14-3-3, a dynein light chain component, tubulin-specific chaperone A and/or a thioredoxin produced by the small-size worms after three hours of incubation. From these Hsp90 and Sj-Ts4 hold the greatest diagnostic promise, as they were also produced by the large size worms at this incubation stage. Finally, complementary immunoproteomics indicates several antigenic proteins, which may help to understand worm-to-worm communication, host-adaptation, and immunogenicity of this parasite.

## Data availability statement

The mass spectrometry proteomics data have been deposited to the ProteomeXchange Consortium via the PRIDE (59) partner repository with the dataset identifier PXD029144.

## Ethics statement

Ethical review and approval was not required for the animal study because the animals used in this study were horses intended for meat production and the animals were slaughtered for human consumption in an approved abattoir. All the activities related to our research were conducted only after the death of the slaughtered animal.

## Author contributions

AN, AS, KH, MN, and KS participated in study design. KH collected live worms needed for the study. KH, KS, AN, and JP conducted the laboratory work at the University of Helsinki. TN provided the LFQ proteomics and statistical analyses. PV conducted the additional perseus/multivariate analysis and KS the STRING PPI analyses. All authors contributed to the article and approved the submitted version.

## Funding

This study was partly supported by grants provided by the Marjatta and Eino Kolli Foundation, Orion Pharma Foundation, Finnish Veterinary Foundation and Finnish Foundation of Veterinary Research. Mass spectrometry-based proteomic analyses were performed by the Proteomics Core Facility, Department of Immunology, University of Oslo/Oslo University Hospital, which is supported by the Core Facilities program of the South-Eastern Norway Regional Health Authority. This core facility is also a member of the National Network of Advanced Proteomics Infrastructure (NAPI), which is funded by the Research Council of Norway INFRASTRUKTUR-program (project number: 295910).

## Conflict of interest

The authors declare that the research was conducted in the absence of any commercial or financial relationships that could be construed as a potential conflict of interest.

## Publisher’s note

All claims expressed in this article are solely those of the authors and do not necessarily represent those of their affiliated organizations, or those of the publisher, the editors and the reviewers. Any product that may be evaluated in this article, or claim that may be made by its manufacturer, is not guaranteed or endorsed by the publisher.
